# CD133 expression in cancer cells predicts poor prognosis of non-mucin producing intrahepatic cholangiocarcinoma

**DOI:** 10.1186/s12967-018-1423-9

**Published:** 2018-03-06

**Authors:** Xiaobo Cai, Jun Li, Xiaodong Yuan, Jingbo Xiao, Steven Dooley, Xinjian Wan, Honglei Weng, Lungen Lu

**Affiliations:** 1 Department of Gastroenterology, Shanghai General Hospital, Shanghai Jiao Tong University School of Medicine, Shanghai, China; 2 Department of Hepatobiliary and Transplant Surgery, University Medical Center Hamburg-Eppendorf, Hamburg, Germany; 3 Department of Medicine II, Section Molecular Hepatology, Medical Faculty Mannheim, Heidelberg University, Mannheim, Germany

**Keywords:** Intrahepatic cholangiocarcinoma, CD133, Epithelial–mesenchymal transition

## Abstract

**Background:**

CD133 is a marker of stem cells as well cancer stem cells. This study investigated the association between CD133 expression in cancer cells and the clinical outcome of non-mucin producing intrahepatic cholangiocarcinoma (ICC).

**Methods:**

Fifty-seven non-mucin producing ICC patients were enrolled in this study. Immunohistochemistry (IHC) and immunofluorescence staining for CD133 as well as other cancer-associated proteins, including cytokeratin 19, TGF-β1, p-Smad2 and epithelial–mesenchymal transition (EMT) markers S100A4, E-Cadherin and Vimentin were analyzed.

**Results:**

IHC staining showed that tumor cells in 52.6% of patients expressed CD133. The CD133^+^ patients had significantly higher metastasis rate than those without CD133^+^ tumor cells (36.7% vs. 10.1%, *p* = 0.03). The CD133^+^ patients had shorter overall and disease-free survival time as compared to the CD133^−^ patients. Furthermore, 90.9% of CD133^+^ patients developed cancer recurrence, as compared to 64.3% of CD133^−^ patients (*p* = 0.02). As compared to CD133^−^ patients, tumor cells in CD133^+^ patients demonstrated high levels of TGF-β/p-Smad2 as well as EMT-like alteration, characterized by loss of E-Cadherin and expression of Vimentin and S100A4.

**Conclusions:**

CD133 expression in ICC tumor cells indicates poor prognosis of the disease and might be associated with TGF-β related EMT alterations.

## Background

Cholangiocarcinoma (CCA) is the second most common hepatic malignancy after hepatocellular carcinoma (HCC) and has a very poor prognosis with the 5-year survival rate < 10% [1]. In terms of the location of the malignancy, CCA can be mainly divided into extrahepatic cholangiocarcinoma (ECC) and intrahepatic cholangiocarcinoma (ICC). In addition, ICC can also be categorized into mucin-producing and mucin-negative. The former arises from large bile duct cells and the latter from small bile duct cells or liver progenitor cells (LPC) [2, 3].

CD133 is a five-transmembrane cell-surface glycoprotein and a marker of stem cells or progenitor cells [4]. It has been used for the identification of cancer stem cells (CSCs) in several types of cancers including CCA [5–7]. CD133 expression is related to poor prognosis of colon cancer and HCC [8, 9]. Two studies showed that CD133 positive CCA had poor prognosis, while another study demonstrated the opposite result [10–12]. How CD133 impacts progression of CCA remains unknown.

Mucing-producing ICC and non-mucin producing ICC exhibit variable biological and clinicopathological features as well as different outcomes. Generally, non-mucin producing ICC is similar to cancer stem cells due to its possible origin from LPC. In this study, we enrolled patients with non-mucin producing and investigated the correlation between CD133 expression and disease prognosis.

## Methods

### Patients and specimens

A total of 59 non-mucin producing ICC patients confirmed by pathologically HE staining who received curative surgery from January 2004 to December 2014 (33 from Shanghai General Hospital, Shanghai Jiaotong University School of Medicine, Shanghai, China and 26 from University Medical Center Hamburg-Eppendorf, Hamburg, Germany) were enrolled. Two patients lost during the follow-up. Hence, 57 patients, 39 men and 18 women, were eventually included in the study. The mean follow-up duration was 25.7 ± 19.1 months. The tumor stage was determined according to the 2009 UICC TNM classification system [13].

The clinical study was approved by the Ethics Committee of Shanghai General Hospital, Shanghai Jiaotong University School of Medicine and the ethics committee of Medical Association of Hamburg. Informed consent was obtained from all participants.

### Histology

Liver tissues were fixed in 4% formaldehyde and embedded in paraffin. 4 μm sections was used for hematoxylin–eosin, Sirius red and immunohistochemistry (IHC) staining. The inflammation grades and fibrosis stages of the peri-tumoral tissues were examined by two experienced pathologists according to the Scheuer scoring system [14].

For IHC, the sections were boiled in 10 mM sodium citrate buffer (pH 6.0) for 10 min for antigen unmasking. After cooling, the sections were incubated in peroxidase blocking reagent (Dako) for 1 h and then incubated with the following primary antibodies at 4 °C for overnight: anti-CK19 (Dako, Hamburg, Germany), 1:200; anti-CD133 (R&D Biotechnology, USA), 1:200; anti-TGF-β1 (Santa Cruz Biotechnology, USA), 1:200; anti-p-Smad2 (Santa Cruz Biotechnology, USA), 1:100; and anti-S100A4 (Sigma-Aldrich Biotechnology, Germany), 1:200; anti-E-Cadherin (1:200; Abcam) and anti-Vimentin (1:200; Abcam). Next day, the sections were incubated at room temperature with the secondary antibody and developed with diaminobenzidine for 5 min.

For semiquantitative analysis, IHC scores were calculated as follows: grade 0, < 1% positive cells; grade 1, ≥ 1% and < 25% positive cells; grade 2, ≥ 25% and < 50% positive cells; grade 3, ≥ 50% and < 75% positive cells; and grade 4, ≥ 75% positive cells. Since p-Smad2 is commonly expressed in tumor cells, we also evaluated the intensity of p-Smad2 staining: grades 1–4: (1) weak positive staining: yellow; (2) moderate positive staining: brown; (3) strong positive staining: deep brown; (4) very strong: black. The final immune staining score for p-Smad2 was calculated as positive area * staining intensity.

For double-fluorescence immunostaining for E-Cadherin and Vimentin, the slides were washed with PBS and incubated with anti-E-Cadherin antibody (1:100; Abcam) at 4 °C overnight. Then, the slides were washed with PBS and incubated with anti-Vimentin antibody (1:100; Abcam) at 4 °C overnight. Next, the slides were incubated with secondary antibodies, Alexa 633 IgG and Alexa 488 IgG (Molecular Probes/Invitrogen, Karlsruhe, Germany) for 30 min at room temperature. The samples were mounted using Dako-Cytomation Fluorescence Mounting Medium. The slides were imaged with a confocal microscope (Leica, Heidelberg, Germany).

### Statistical analysis

Data were analyzed using the SPSS version 13.0 for Windows (SPSS Inc, Chicago, IL, USA), and are presented as means and standard deviations (± SD). Student’s t-test was used to compare the continuous quantitative data. A two-tailed Wilcoxon signed rank test was used to compare ranked variables. The Kaplan–Meier analysis was applied to evaluate overall and disease-free survival, and different groups were compared with the log-rank test. *p* < 0.05 was considered to be statistically significant.

## Results

### CD133 expression in ICC

**Fig. 1:**
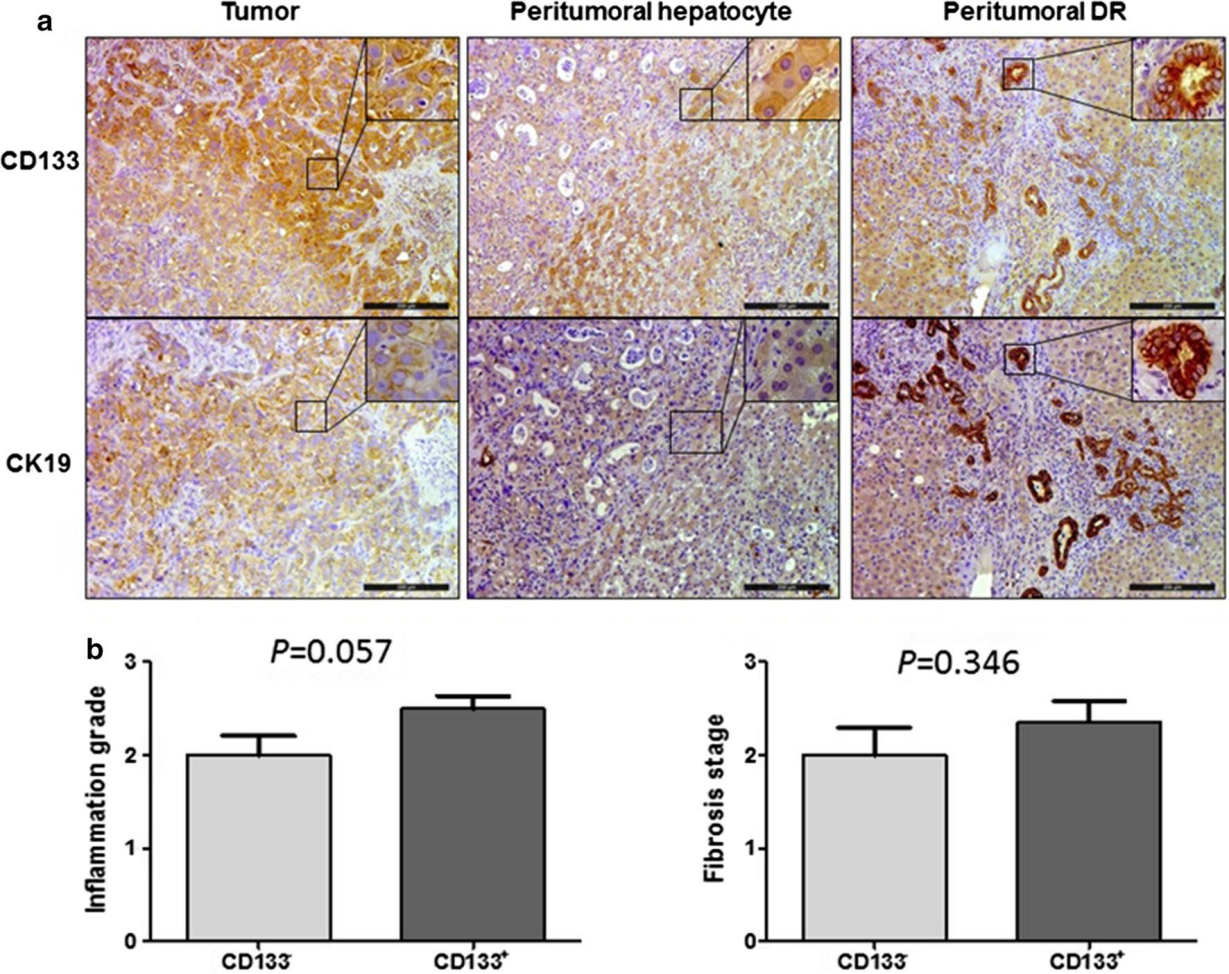
CD133 expression in tumor and peri-tumoral tissues. **a** CD133 expressed in tumor cells, peri-tumoral hepatocytes and ductular cells. **b** The relationship between CD133 expression in tumor and peri-tumoral liver inflammation and fibrosis

IHC staining showed that 30 patients (52.6%) had CD133 expression on the membrane of tumor cells. Besides cancer cells, CD133 was also expressed in peri-tumoral ductular reaction (DR). Interestingly, hepatocytes in two patients also expressed CD133 (Fig. 1a). Furthermore, we analyzed the relationship between CD133 expression and liver inflammation and fibrosis. CD133^+^ ICC displayed more severe liver inflammation and fibrosis in peri-tumoral areas than CD133^−^ ICC, although the difference was not statistically significant (*p* = 0.056 and 0.06, respectively, Fig. 1b).

### ICC patients with CD133 expression had poor prognosis

**Fig. 2:**
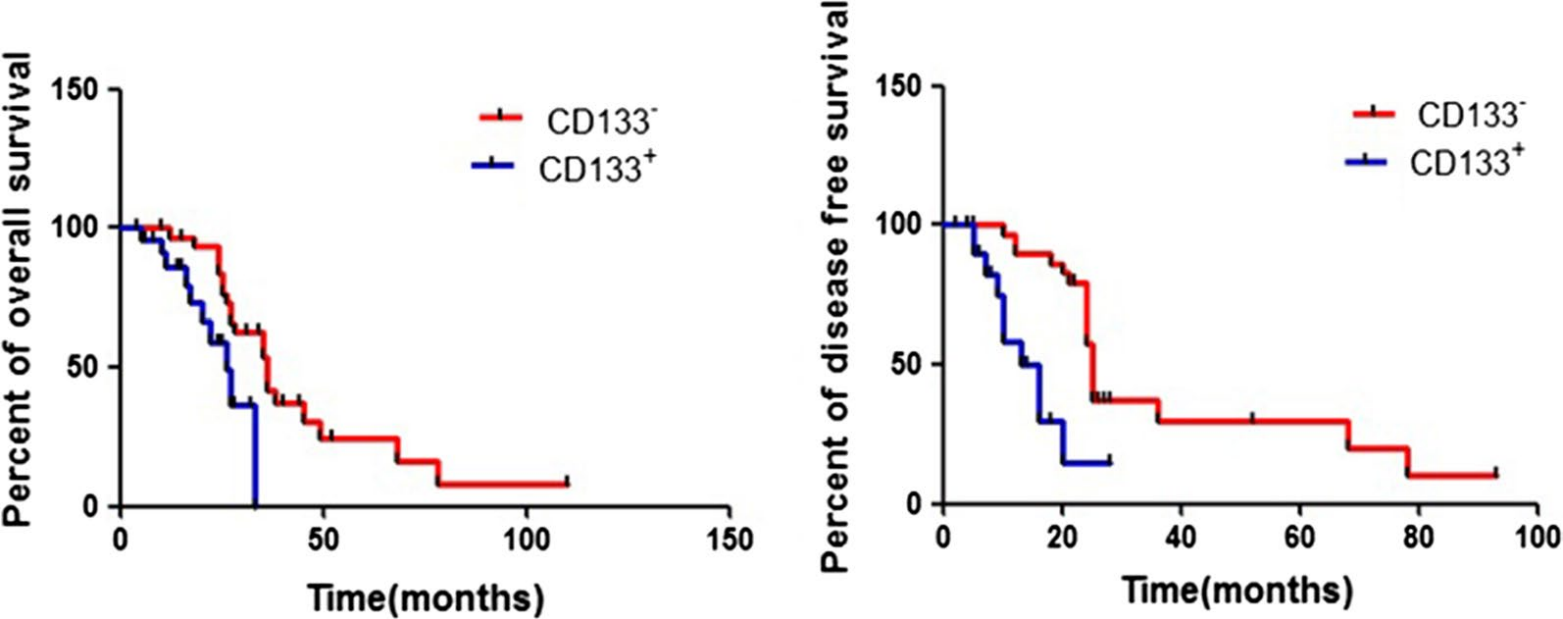
Survival analysis showed that overall and disease-free survival (DFS) were higher in the CD133 negative group than in the CD133 positive group

Next, we analyzed clinical outcome of ICC patients according to CD133 expression in tumor cells. The CD133^+^ patients had significantly higher metastasis rate than CD133^−^ patients at the time of diagnosis (36.7% vs. 10.1%, *p* = 0.03). We followed up these patients for 25.7 ± 19.1 months and found that 90.9% of CD133^+^ patients had ICC recurrence, as compared to 64.3% of CD133^−^ patients(*p* = 0.02, Table 1). Survival analysis showed shorter overall and disease-free survival rates in CD133^+^ patients as compared to CD133^−^ patients (Fig. 2). These results suggested that CD133 expression was related to poor prognosis in non-mucin producing ICC patients.

**Table 1:** Comparison of clinicopathological parameters between CD133^−^ and CD133^+^ ICC patients

	CD133^−^ (n = 27)	CD133^+^ (n = 30)	*P*
Age	57.4 ± 8.4	60.5 ± 11.5	0.47
Gender			0.76
Male	19 (70.3%)	20 (66.7%)	
Female	8 (29.7%)	10 (33.3%)	
TNM stages			
T			0.37
1–2	14 (51.9%)	12 (40.0%)	
3–4	13 (48.1%)	18 (60.0%)	
N			0.41
0	19 (70.4%)	18 (60.0%)	
1	8 (29.6%)	12 (40.0%)	
M			0.03
0	24 (88.9%)	19 (63.3%)	
1	3 (10.1%)	*11 (36.7%)*	
Differentiation			0.89
1–2	14 (51.9%)	15 (50.0%)	
3–4	13 (48.1%)	15 (50.0%)	
Recurrence			0.02
0	10 (35.7%)	3 (10.0%)	
1	18 (64.3%)	*27 (90.9%)*

### CD133^+^ cancer cells demonstrated EMT markers

**Fig. 3:**
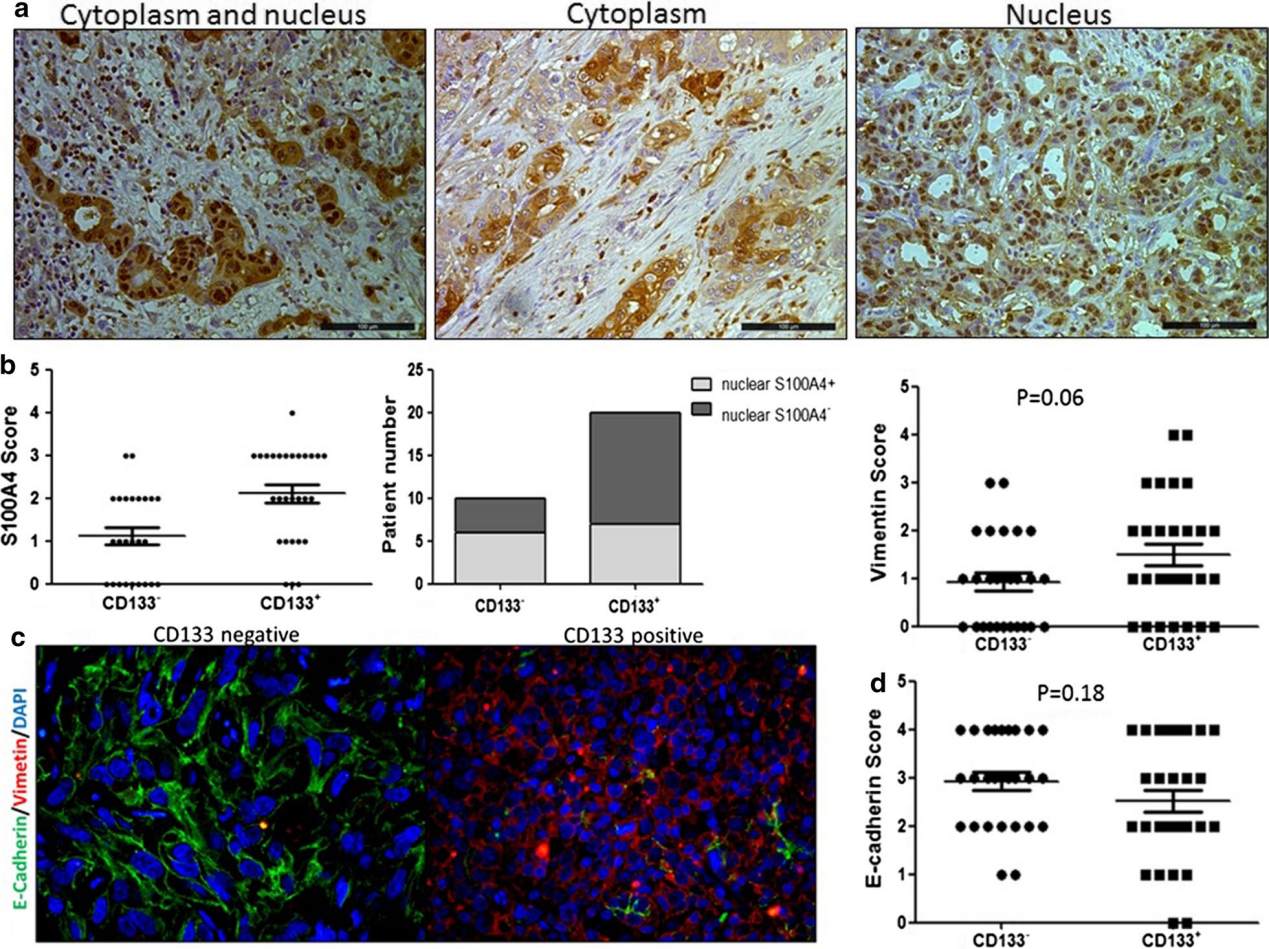
CD133^+^ tumor cells showed EMT phenotype. **a** S100A4 expression in tumor cells. **b** Expression of total S100A4 and nuclear S100A4 was higher in the tumors of CD133^+^ patients than in CD133^−^ patients. **c** A representative patient with CD133 positive expression showed loss of E-Cadherin and expression of Vimentin as well as altered cell shape. **d** IHC staining showed the trend of higher expression of Vimentin and less expression of E-Cadherin in CD133^+^ patients than CD133^−^ patients

Given that tumor metastasis is closely associated with EMT, we performed IHC staining for S100A4, a marker of EMT, in liver tissue samples. CD133^+^ patients had higher levels of S100A4 in tumor cells than CD133^−^ patients (2.10 ± 0.21 vs. 1.12 ± 0.20, *p* = 0.001). S100A4 protein was localized in the cytoplasm, nucleus, or both. More patients had nuclear expression of S100A4 in the CD133^+^ group, although without statistical significance (65% vs. 40%, *p* = 0.193 Fig. 3a, b).

To further verify the relationship between EMT and CD133 expression, we performed fluorescence co-immunostaining for E-Cadherin, an epithelial marker, and Vimentin, a mesenchymal marker, in CD133 positive and negative patients, respectively. As compared to CD133^−^ tumor cells, CD133^+^ ICC lost epithelial marker E-Cadherin and acquired mesenchymal marker Vimentin, indicating an EMT-like alteration in these tumor cells (Fig. 3c). IHC staining showed the trend of higher expression of E- cadherin and lower expression of Vimentin in CD133^−^ ICC than in CD133^+^ patients(*p* = 0.18 and *p* = 0.06, respectively) (Fig. 3d).

**Fig. 4:**
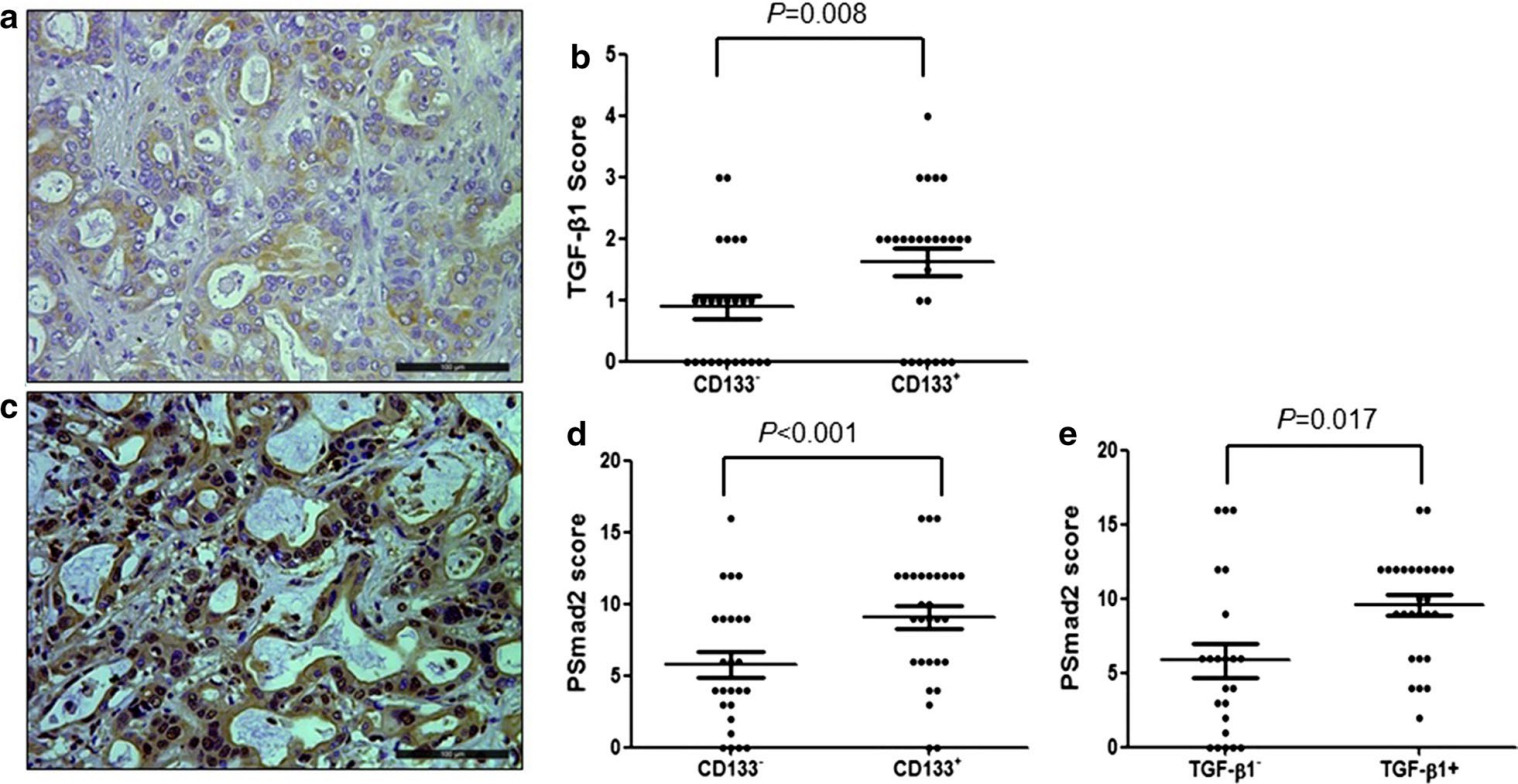
The relationship between CD133 expression and TGF-β1 signaling in tumor cells. **a** TGF-β1 expression in ICC cells. **b** CD133^+^ ICC cells had strong TGF-β1 expression; **c** p-Smad2 was expressed by CCA cells. **d** CD133^+^ ICC cells had robust p-Smad2 expression. **e** TGF-β1^+^ CCA had higher scores of p-Smad2 expression in tumor cells

Given a crucial role of TGF-β1 in EMT, we investigated the expression of TGF-β1 and its downstream protein p-Smad2 by IHC. Expression of TGF-β1 in tumor cells was seen in most ICC patients (Fig. 4a). However, CD133^+^ patients displayed higher levels of TGF-β1 expression in tumor cells than CD133^−^ patients (*p* = 0.017, Fig. 4b). Consistent with high levels of TGF-β1, CD133^+^ tumor cells also expressed higher levels of p-Smad2 than CD133^−^ tumor cells (p = 0.008, Fig. 4c, d).

## Discussion

Several clinical studies had investigated the relationship between CD133 expression and clinical outcome of cholangiocarcinoma (Ref.). However, the results are controversial. Shimada and colleagues showed that CD133 expression in tumor cells was an indicator of poor prognosis for ICC patients. The 5-year survival rate was lower in CD133^+^ patients than in CD133^−^ patients (8% vs. 57%) [10]. Another study with 34 ICC and perihilar CC patients also demonstrated that strong expression of CD133 in tumor was related to nodal metastasis and positive surgical margin status. Furthermore, CD133^+^ cells had a higher invasive ability in vitro [11]. However, Fan et al. study with 25 ICC and 29 perihilar CC patients displayed opposite result [12], wherein positive expression of CD133 in tumor cells was correlated with high or moderate tumor differentiation and predicted better prognosis of the disease. Notably, these studies included different subtypes of CCA and did not focus on ICC. ICC is divided into two groups according to the presence or absence of mucin products [2]. The mucin-producing ICC arises from large bile duct epithelial cells similar to perihilar or extrahepatic CC, while the non-mucin producing ICC arises from small bile ducts or LPC. A study with 87 cases of ICC demonstrated that ICC from large ducts had significantly higher incidence of perineural invasion, lymph node metastasis, vascular invasion, intrahepatic metastasis and recurrence as well as worse survival as compared to ICC from small ducts [15]. Therefore, the role of CD133 in different pathological types of CCA might be different.

The current study focused on the relationship between CD133 expression and non-mucin producing ICC. We found that more than 50% of non-mucin producing ICC patients expressed CD133 in tumor cells. These CD133^+^ patients had higher metastasis and recurrence rate after surgery and worse overall and disease-free survival. These results suggested that CD133 is an indicator for poor prognosis of non-mucin producing ICC.

CD133 is thought to be a marker of CSC also known as tumor-initiating cells, which are responsible for metastasis, chemotherapy resistance, and tumor recurrence [16]. CSC exhibit EMT phenotype that facilitate tumor metastasis and recurrence. Thus, we investigated the relationship between CD133 expression and tumor EMT. We examined the expression of an EMT marker S100A4 in ICC tissue samples. CD133^+^ patients had higher total S100A4 and nuclear S100A4 expression. Fabris et al. showed that nuclear expression of S100A4 by CCA tumor cells was a strong predictor of metastasis and poor survival after resection by increasing CCA cell motility, invasiveness, and MMP-9 secretion [17]. These findings partially explain why CD133^+^ ICC patients have poor prognosis. Next, we performed immunostaining for epithelial marker E-Cadherin and mesenchymal marker Vimentin and found that CD133^+^ ICC cells underwent EMT-like alteration characterized by lower E-Cadherin and higher Vimentin expression, indicating that EMT might be a mechanism linking CD133 expression with poor prognosis.

TGF-β1 is a key cytokine for inducing EMT, which contributes to tumor metastasis and recurrence [18]. Therefore, we evaluated the relationship between CD133 expression and activation of TGF-β1 signaling in ICC. As expected, CD133^+^ ICC displayed both higher levels of TGF-β1 and p-Smad2 expression. These results suggested a potential link between enhanced TGF-β1–Smad2 signaling and CD133 expression in non-mucin producing ICC.

Our study demonstrated that CD133 expression in tumor cells is an indicator for poor prognosis of non-mucin producing ICC. CD133 expression in tumor cells might be associated with TGF-β1-p-Smad2-EMT axis in ICC. The underlying detailed mechanisms require further investigation in the future.

## Conclusions

Our current work is the first attempt to evaluate the role of CD133 expression in tumor cells for the prognosis of non-mucin producing ICC, providing additional insights on the molecular events responsible for prognosis prediction and antitumor potency.
